# Tactile bill-tip organs in seabirds suggest conservation of a deep avian symplesiomorphy

**DOI:** 10.1098/rsbl.2024.0259

**Published:** 2024-09-18

**Authors:** Carla J. du Toit, Alexander L. Bond, Susan J. Cunningham, Daniel J. Field, Steven J. Portugal

**Affiliations:** ^1^ Department of Earth Sciences, University of Cambridge, Cambridge CB2 3EQ, UK; ^2^ FitzPatrick Institute of African Ornithology, University of Cape Town, Rondebosch 7700, South Africa; ^3^ Department of Biological Sciences, University of Cape Town, Rondebosch 7700, South Africa; ^4^ Bird Group, Natural History Museum, Akeman Street, Tring, Hertfordshire HP23 6AP, UK; ^5^ University Museum of Zoology, University of Cambridge, Cambridge CB2 3EJ, UK; ^6^ School of Biological Sciences, Royal Holloway University of London, Egham, Surrey TW20 0EX, UK; ^7^ Department of Biology, University of Oxford, Oxford OX1 3SZ, UK

**Keywords:** seabirds, sensory ecology, tactile, comparative morphology, foraging

## Abstract

Birds’ bills are their main tactile interface with the outside world. Tactile bill-tip organs associated with specialized foraging techniques are present in several bird groups, yet remain understudied in most clades. One example is Austrodyptornithes, the major seabird clade uniting Procellariiformes (albatrosses and petrels) and Sphenisciformes (penguins). Here, we describe the mechanoreceptor arrangement and neurovascular anatomy in the premaxillae of Austrodyptornithes. Using a wide phylogenetic sample of extant birds (361 species), we show that albatrosses and penguins exhibit complex tactile bill-tip anatomies, comparable to birds with known bill-tip organs, despite not being known to use tactile foraging. Petrels (Procellariidae, Hydrobatidae and Oceanitidae) lack these morphologies, indicating an evolutionary transition in bill-tip mechanosensitivity within Procellariiformes. The bill-tip organ in Austrodyptornithes may be functionally related to nocturnal foraging and prey detection under water, or courtship displays involving tactile stimulation of the bill. Alternatively, these organs may be vestigial as is likely the case in most palaeognaths (e.g. ostriches and emu). Ancestral state reconstructions fail to reject the hypothesis that the last common ancestor of Austrodyptornithes had a bill-tip organ; thus, tactile foraging may be ancestral for this major extant clade, perhaps retained from a deeper point in crown bird evolutionary history.

## 1. Introduction

The bills of birds act as the main tactile interface with their surroundings, analogous to the hands of primates. This is reflected in bill anatomy, as avian bill tips contain numerous mechanoreceptors (predominantly Herbst corpuscles), usually located in the dermal layers between the bone and rhamphotheca [1,2]. Some taxa are more reliant on their bills as tactile sensors than others and have evolved specialized bill-tip organs [1–7]. These bill-tip organs have been linked to foraging behaviours relying on tactile sensation: parrots (order: Psittaciformes) use their bills for fine-scale manipulation of food items [7,8]; various Anseriformes (ducks and kin) use dabbling and filter feeding to extract food particles from mud or water [1,6,9–11]; and various probe-foraging birds (ibises, kiwis and scolopacid shorebirds) can sense vibrational cues in mud and water to locate invertebrate prey at a distance from their bills, a sensory modality known as remote touch [2–4,12–16]. These bill-tip organs contain large numbers of mechanoreceptors organized into distinct units within the distal tip of the bill [1–4,6,7,10,17]. Large numbers of sensory pits (external openings of neurovascular foramina) on the distal surfaces of the beak bones are characteristic of the bill-tip organs seen in Anseriformes and remote-touch probing birds [2]. Species that use tactile-foraging methods also show hypertrophy of the brain regions responsible for processing tactile information from the bill [18,19].

The evolution of these bill-tip organs remains largely unstudied. It has been hypothesized that remote-touch capability evolved convergently three times among crown birds [17], though its evolutionary history has only been studied in palaeognathous birds (ostriches and kin). The bill-tip organ is plesiomorphic for crown group palaeognaths, seemingly inherited from a previous common ancestor which used remote-touch probing, and is likely vestigial in all extant palaeognaths except kiwi (Apterygidae) [2]. The evolutionary origins of bill-tip organs in other birds (including parrots, Psittaciformes; and waterfowl, Anseriformes) remain unclear. Our knowledge of the distribution of bill-tip organs may also be incomplete, as anatomical studies have generally focused on the aforementioned groups, leaving wide phylogenetic gaps in our understanding of the prevalence of bill-tip organs among crown birds.

One major clade awaiting detailed examination of bill-tip morphology is Austrodyptornithes, the clade of seabirds comprising Sphenisciformes (penguins) and Procellariiformes (albatrosses and petrels) [20]. Most of these taxa are not known to use tactile-foraging techniques, with the exception of some prions and fulmars (Procellariiformes: Procellariidae), which filter food through lateral lines of lamellae along the edges of their premaxillae [21–26]. Austrodyptornithes feed on marine animals and forage in pelagic waters, only returning to land to breed [27], so observing how they locate prey is challenging. Studies of their foraging behaviour rely predominantly on dietary composition (e.g. from stomach contents) [28] and the use of remote tracking devices [29]. Most species have good vision, and prey capture is generally assumed to be visually guided [30–35], although Procellariiformes can locate accumulations of prey from several kilometres away using olfaction [36,37]. Many species hunt in low-light levels (e.g. at night or while diving deep underwater) [27,38]. When the eyes are submerged underwater, the birds lose binocularity at the tip of their bill, and spatial resolution decreases with increasing water depth [30,32,33]; therefore, tactile sensitivity may be beneficial during foraging for the location and selection of prey.

**Figure 1.**
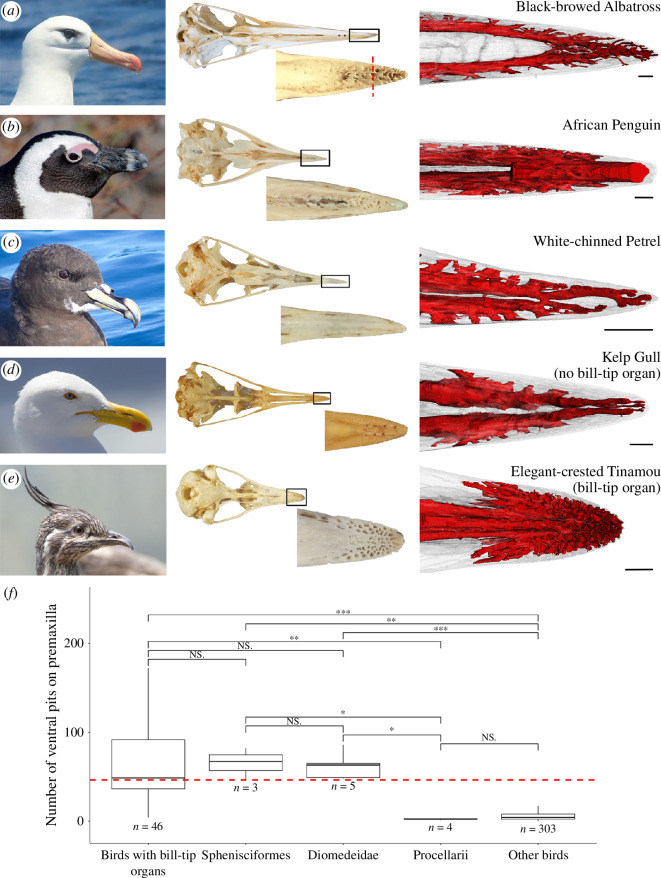
(*a–e*) The bills of five bird species: three Austrodyptornithes, (*a*) black-browed albatross (*Thalassarche melanophris*); (*b*) African penguin (*Spheniscus demersus*); and (*c*) white-chinned petrel (*Procellaria aequinoctialis*); a bird without a bill-tip organ, (*d*) kelp gull (*Larus dominicanus*); and a bird known to have a bill-tip organ, (*e*) elegant-crested tinamou (*Eudromia elegans*). *Left:* photographs of each species (credit: du Toit & Field). *Middle:* ventral view of the skull, with zoomed in insets of the distal tips of the premaxilla, showing interspecific differences in external bone morphology. *Right:* ventral views of µCT scan-generated three-dimensional models of the distal tips of the premaxillae. Red: endocasts of the neurovascular canals; Grey: bone. See electronic supplementary material S1 for animations. Scale bars: 1.5 mm. (*f*) Box plot showing variation in number of ventral pits between Sphenisciformes, Procellariiformes (split into albatrosses and Procellarii), birds with known bill-tip organs and all other birds. The dashed red line indicates the estimated trait value for ancestral Austrodyptornithes (REML estimate). ‘*n*’ = number of species. Asterisks indicate statistically significant differences from two-sided Mann–Whitney *U*-tests, *p*-values: *** < 0.001; ** < 0.01; * < 0.5; ‘NS.’ > 0.05. See electronic supplementary material, table S3 for test statistics.

Here, we report unusual morphologies in the premaxillae of Austrodyptornithes, specifically in penguins (Sphenisciformes) and albatrosses (Procellariiformes: Diomedeidae). These clades exhibit large numbers of sensory pits on the ventral surfaces of their premaxillary bones (figure 1*a–e*). We hypothesize that these unusual morphologies may represent a tactile bill-tip organ in Austrodyptornithes. We place our findings in phylogenetic context using a representative sample of all extant birds and compare austrodyptornithine premaxillae to birds with known bill-tip organs to outline hypotheses regarding the function and evolutionary history of the observed features. To further investigate the presence of bill-tip organs in Austrodyptornithes, we compare the internal neurovascular anatomy and soft tissue histology of their premaxillae to birds with and without bill-tip organs. Finally, we discuss the implications of our findings for understanding of the ecology of Austrodyptornithes and the evolution of avian tactile systems on a broad phylogenetic scale.

## 2. Methods

### (a) Sample and measures of sensory pitting on premaxillary bones

Our sample comprised 361 species of extant birds, representing 84% of extant family-level clades [39] and all 41 extant order-level clades (electronic supplementary material, table S1, taxa sampled). Bill length was measured as the distance from the distal tip of the premaxilla to the craniofacial hinge. Bill-tip anatomy/anatomies refer to the anatomical structures found in birds beaks, regardless of the presence or absence of a bill-tip organ (the structure made up of both bone and soft tissues). All measures of sensory pits were taken from photographs of skulls in ventral view, using ImageJ2 [40]. An established workflow was used to extract the relevant measurements from photographs [41]: pits on the distal 20% of the premaxilla (bill length divided by five) were counted, as this is the region shown to represent the greatest variation in sensory pitting [2]. Two measures of these pits were taken for each specimen: number of sensory pits and average nearest neighbour distance between the pits (using the ImageJ plugin, *NND* [42]; pits were selected by threshold values, and any errors/missing pits were then manually removed/selected).

### (b) Reconstructing endocasts of internal neurovasculature

Specimens from four austrodyptornithine taxa were µCT scanned to visualize differences in the three-dimensional internal neurovasculature of the premaxilla: a penguin (Spheniscidae), *Spheniscus demersus*; an albatross (Diomedeidae), *Thalassarche melanophris*; and two petrels (Procellariidae and Hydrobatidae, respectively), *Puffinus lherminieri* and *Hydrobates pelagicus*. To provide comparative examples of bird bills with and without bill-tip organs [2], a tinamou (*Eudromia elegans*) and a gull (*Larus fuscus*) were scanned. Skulls from the University of Cambridge Museum of Zoology were scanned using a Nikon 49 Metrology XT H 225 ST high-resolution CT scanner at the Cambridge Biotomography Centre. The upper bill was scanned from the distal tip to the craniofacial hinge, with voxel sizes of 14–18 µm to allow for distinction of the foramina and canals within the bone [5].

Visualization and segmentation of the scans were performed in *VGSTUDIOMAX* V2023.2.1 (Volume Graphics). The premaxillary bone was segmented from the distal tip to the distal opening of the nares. Endocasts of the soft tissue spaces within the bones were segmented (based on threshold grey values), and the neurovascular canals were manually isolated following the paths from the large central canals to the foramina/pits on the surface of the bone. Similar methodologies have previously been used to isolate endocasts of neurovasculature and other soft tissues from the negative spaces in bone from µCT scans [43–45].

### (c) Soft tissue histology

We sectioned premaxillae from four species of Austrodyptornithes: two albatrosses (*Diomedea exulans* and *T. melanophris*), one petrel (*Procellaria aequinoctialis*) and one penguin (*S. demersus*). All samples were sourced off the coast of the Western Cape Province of South Africa, from birds that died from causes unrelated to this study (mostly as accidental bycatch from long-line fisheries). The specimens were frozen for several days before preparation. Established workflows for bird beak soft tissue histology were used to prepare, section and stain specimens [2,4,5,17] (see electronic supplementary material for details). Slides were imaged using a Leica Z16 monozoom microscope and attached GXcam camera, with GXCapture-T software.

### (d) Phylogenetic and statistical analyses

Two-sided Mann–Whitney *U*-tests were performed using the R [46] package *ggsignif* [47] to assess variation in numbers of sensory pits on the ventral surfaces of the premaxillae between bird groups (Sphenisciformes, Procellariiformes (split into albatrosses and Procellarii), birds with known bill-tip organs and all other birds). Boxplots were generated using *ggplot2* [48].

The species-level phylogeny and branch length estimates used were based on a previously published tree [49], with the exception of topologies for Palaeognathae and Procellariiformes, for which we used time-calibrated topologies [50] from the phylogeny, which provided the phylogenetic backbone for the species-wide tree [49]. The original species-level phylogeny consisted of 9993 species (utilizing a previous dataset [51]), which we pruned down to our 361 species using the *ape* [52] package. Ancestral state estimates and 95% confidence intervals based on tip values and estimated branch lengths were generated from our species-level phylogeny using the package *ape* [52] (*ace* function; REML (restricted maximum likelihood) method.

The species-level phylogeny was collapsed to the level of orders and families [39], and larger clades (electronic supplementary material, table S2, clade definitions; e.g. Inopinaves, the potential clade uniting Telluraves (the large clade comprising most traditionally defined ‘higher land-birds’), with the hoatzin (*Opisthocomus hoazin*) [50]). Plotting continuous morphological variables was done using the package phytools [53]. The shaded region on our phylomorphospace was drawn in Adobe Photoshop (2018), including all taxa with known bill-tip organs [2] while minimizing the number of vertices.

## 3. Results

### (a) Phylogenetic variation in sensory pitting on the ventral premaxilla

Birds known to have bill-tip organs have significantly greater numbers of pits on the ventral surfaces of their premaxillae than species without bill-tip organs (‘Other birds’, figure 1*f*; electronic supplementary material, table S3). The number of pits on the premaxillae of penguins and albatrosses are not significantly different from each other or from taxa with known bill-tip organs, but they are significantly greater than birds without bill-tip organs and Procellarii (non-albatross Procellariiformes). Procellarii have significantly fewer pits than birds with bill-tip organs and are not significantly different from birds without bill-tip organs (figure 1*f*; electronic supplementary material, table S3).

The ancestral state estimate for the number of ventral pits on the premaxilla of the last common ancestor of Austrodyptornithes overlaps with the measured range in extant birds with bill-tip organs and is greater than in Procellarii and other birds (figure 1*f*).

**Figure 2.**
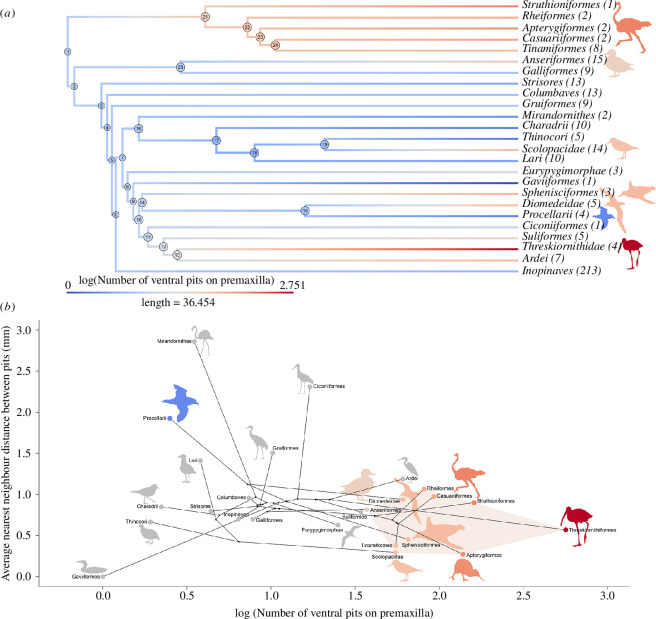
Data on sensory pitting on the ventral surface of the premaxillary bones of modern birds (361 species, all 42 orders). Ancestral trait values are REML (restricted maximum likelihood) estimates from species-level phylogenies. (*a*) Phylogeny of major bird clades, with branch lengths representing timescale in millions of years [49,50]. Colours represent the spectrum of numbers of ventral pits, showing average values for clades at the tips and the estimated ancestral states at the nodes. Numbers in brackets indicate numbers of species sampled. (*b*) Phylomorphospace of major bird clades, showing the relationship between number of ventral pits and nearest neighbour distance between pits. The shaded region shows the morphospace of birds with known bill-tip organs. Colours are used to highlight clades of interest and the morphospace of birds known to have bill-tip organs.

The number of ventral pits (log-transformed) on the premaxilla in extant birds and estimated ancestral states is mapped onto phylogenies as colour gradients (figure 2*a*; electronic supplementary material, figure S1 for species-level tree). Birds with known bill-tip organs (palaeognaths, anseriforms, threskiornithids and scolopacids), penguins and albatrosses all exhibit distinctly greater numbers of pits than other birds (indicated by red colours), including Procellarii. Suliformes and Ardei (non-ibis Pelecaniformes) also stand out with respect to this character, and the phylogenies illustrate multiple examples of large numbers of ventral pits in Aequorlitornithes (clade branching from node 7, figure 2*a*), potentially indicating the presence of previously undocumented bill-tip organs in this group.

Extant albatrosses and penguins fall within the distribution of birds with known bill-tip organs in the morphospace characterized by large numbers of pits clustered closely together (figure 2*c*, shaded region). Procellarii fall well outside this morphospace, exhibiting low numbers of pits spaced close together, with the most similar group being Lari (gulls and kin, figure 2*b*).

Ancestral state estimates for the last common ancestors of Procellariiformes (log number pits: 0.86 ± 0.45; NND (nearest neighbour distance): 1.13 ± 0.54 mm) and all Austrodyptornithes (log number pits: 1.09 ± 0.33; NND: 0.92 ± 0.40 mm) both fall outside the bill-tip organ morphospace, indicating low numbers of pits that were relatively closely spaced on their premaxillae.

### (b) Variation in three-dimensional morphology of neurovascular canals and soft tissue histology

Procellarii (non-albatross Procellariiformes) most closely resemble birds without bill-tip organs (example of a gull used, figure 1*d*) in terms of the three-dimensional architecture of the neurovascular canals within the bone (figure 1*c*; electronic supplementary material S1 for three-dimensional animations). They possess two lateral canals containing superior branches of the premaxillary nerve [10] and associated vasculature (median palatine vessels [54]; electronic supplementary material, figure S2a). The albatross is superficially similar, but the branching pattern of the canals at the tip of the bill is more complex and shows a greater density of neurovascular canals (figure 1*a*). The penguin (figure 1*b*) is intermediate between albatrosses and birds with bill-tip organs (example of a tinamou used; figure 1*e*): along with the two canals containing the superior branches of the premaxillary nerve, the endocast shows the foramina on the ventral surface of the bone as also being connected to the inferior branch [10] of the premaxillary nerve (as seen in the tinamou). In the gull and Procellariiformes, an endocast of the inferior branch cannot be segmented, as it is not enclosed by bone. Instead, it runs along the medial line of the palate in Procellariiformes and does not innervate the tissues within the neurovascular foramina (electronic supplementary material, figure S2a).

**Figure 3.**
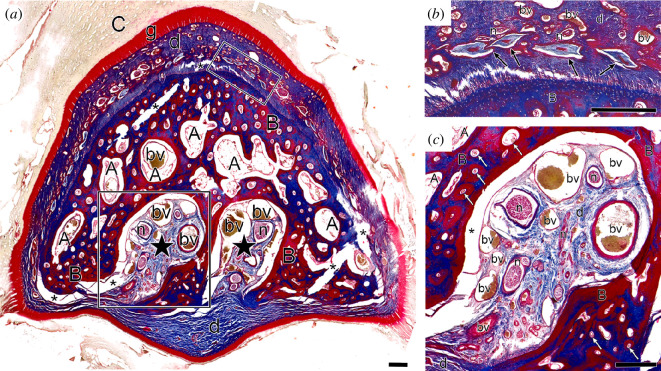
Histological coronal section of the premaxillary tip of a wandering albatross (*Diomedea exulans*), stained with Masson’s trichrome. Relative position is shown by the red-dotted line in figure 1*a*. (*a*) Entire section of the premaxilla, showing positions of two large neurovascular foramina (black stars) in the bone (B) layers of the rhamphotheca (cornified (C) and germinative (g)), deposits of adipose (A) and larger nerves (*n*; branches of *R. premaxillaris* [54]) and median palatine blood vessels (bv). The positions of (*b–c*) are indicated by black boxes. (*b*) Part of the sheet of Herbst corpuscles (black arrows) in the dermis (d) which surround the bone of the premaxillae. (*c*) A single neurovascular canal opening onto the ventral surface of the premaxillary bone—note the absence of Herbst corpuscles. White arrows indicate vascular canals in bone. Asterisks indicate standard artefacts of sectioning. Scale bars: 0.5 mm.

While the canals in the premaxillary bone contain neurovascular tissues, there are other large cavities within the bone (electronic supplementary material, figure S1) containing deposits of adipose tissue (figure 3*a*; electronic supplementary material, figure S2a). The bone itself is well vascularized, with multiple canals containing blood vessels throughout the bone (figure 3*c*; electronic supplementary material, figure S2a,c). All sampled representatives of Austrodyptornithes have a sheet of Herbst corpuscles in the dermis surrounding the dorsal and lateral surfaces of the premaxilla, with very few corpuscles in the ventral dermis (figure 3*b*; electronic supplementary material, figure S2a,b). Sparse Herbst corpuscles can be seen in the mediopalatal ridge of the petrel (electronic supplementary material, figure S2*a*). In the albatrosses, the numerous neurovascular foramina on the ventral surface of the premaxilla do not contain Herbst corpuscles (figure 3*c*). In all Austrodyptornithes sectioned, Herbst corpuscles are clustered in the foramina on the dorsal and lateral surfaces of the bone (electronic supplementary material, figure S2).

## 4. Discussion

Albatrosses and penguins have high densities of neurovascular foramina in a localized region on the distoventral surface of their premaxillae, like birds with known tactile bill-tip organs. These ventral foramina do not contain Herbst corpuscles, although these corpuscles are present within foramina on the other surfaces of the premaxilla. Such complex arrangements of mechanoreceptors and high densities of neurovascular foramina in the bill are generally characteristics of taxa employing specialized tactile-foraging techniques [2–7]. Our data show that Austrodyptornithes exhibit intraclade variation: Procellarii (non-albatross Procellariiformes) exhibit a reduced number of canals related to neurovascular tissues on the ventral surfaces of their distal premaxillae compared to albatrosses, penguins and birds with known bill-tip organs. Our observations therefore suggest that albatrosses and penguins possess some forms of tactile bill-tip organ, which is absent or greatly reduced in Procellarii. Other groups of birds with known bill-tip organs show high interspecific variation in their bill-tip organs: the extent of pitting and density of Herbst corpuscles in the bills of waterfowl (Anseriformes) and ibises (Threskiornithidae) are related to the habitats in which they forage [5,17], and their use of different foraging techniques [55]; scolopacid shorebirds show significant variation in bill-tip organ morphology in relation to foraging ecology [3,13,56], and a vestigial bill-tip organ is likely present in ruddy turnstones (*Arenaria interpres*) [2], which do not employ tactile foraging [57]. Non-probing representatives of Palaeognathae show reduction in their bill-tip organs compared to kiwi, which employ remote-touch probing while foraging [2,4,58,59].

### (a) Functional significance of bill-tip organs in penguins and albatrosses

Almost all birds with known bill-tip organs use them for specialized tactile foraging [1–8]. Neither albatrosses nor penguins have been documented to rely on tactile stimuli during foraging [27,38], although questions regarding how they locate prey underwater remain unanswered [60]. Tactile sensation may be useful when foraging in low-light levels, such as at night or when diving [30,32–34,61]. Although the eyes of penguins and albatrosses are specialized to improve vision underwater and in low light, they lose areas of binocular vision (particularly at the tip of their bill) and spatial resolution underwater, an effect that increases with water depth [30,32,33,35,62]. Therefore, initial prey location may be visual, but the final capture and grasping of slippery, fast-moving prey (e.g. fish or squid) may be aided using tactile cues. Indeed, seals rely on tactile information (via their whiskers) while diving and hunting for prey in low light [63–65], indicating a functionally analogous sensory system in mammals. The bill-tip organ may also be used for non-foraging behaviours. Many Austrodyptornithes have highly tactile courtship displays, in which males and females touch and rub their bills against each other [27]. While we consider it unlikely that this behaviour explains the origin of the bill-tip organ in Austrodyptornithes, we suggest the evolution of these elaborate courtship displays may be related to the retention of plesiomorphic, highly touch-sensitive bills. Although these functional interpretations are compelling, they are at odds with the observation that Procellarii lack bill-tip organs, as many use similar foraging strategies to albatrosses [27,38], and almost all have highly tactile courtship displays [27]. Indeed, various fulmars and prions use tactile-foraging techniques (filter-feeding using rows of overlapping lamellae within their bills) [21–26,34]. However, the lack of high densities of neurovascular foramina on the distal portions of their premaxillae is expected in these taxa, as the lamellae are located on the proximal portions of their bills.

Experimental sensory assays, as have been used on remote-touch foraging birds and ducks [12,14,66–68], may be necessary to clarify whether Austrodyptornithes use tactile cues during foraging and could be used to determine whether bill-tip sensitivity differs among different species within the clade. If these organs prove to be largely non-functional, the presence of a bill-tip organ may be essentially vestigial, as is likely the case in all non-kiwi palaeognaths [2]. This may be the case as many of the foramina on the ventral surface of the premaxillae of Austrodyptornithes do not contain Herbst corpuscles, potentially indicating a lack of mechanosensory function. Future work investigating neurovascular structures on the mandibles may also provide more insight. Compared to kiwi and other tactile-foraging birds, other palaeognaths lack hypertrophy in the brain regions which process tactile information from their bills [19]. Comparing the same regions of the brain for Austrodyptornithes could clarify whether they process high volumes of sensory information from their bills. Detailed examinations of the bill tips of early fossil austrodyptornithines would improve confidence in our ancestral state reconstructions [69,70] and could help shed light on whether the apparent bill-tip organs in penguins and albatrosses are vestigial with respect to the plesiomorphic condition for the clade.

Beyond shedding light on the evolution of tactile-foraging ecology among extant birds, these findings may have conservation implications: the tactile cues used to locate or select prey may play a role in determining why some seabird taxa are more prone to consuming plastic waste than others [71–73] or why some are more likely to become bycatch victims of long-line and gillnet fishing [74–76]. These are fundamental problems facing the conservation of Austrodyptornithes on a global scale, most of which are threatened [71], and aspects of their foraging and sensory ecology should not be overlooked when considering mitigation factors [60,77].

### (b) Evolution of tactile bill-tip organs in Austrodyptornithes and beyond

Given the similar morphologies present in both albatrosses and penguins, the bill-tip organ may be plesiomorphic in Austrodyptornithes, implying an apomorphic reduction of this organ in Procellarii. This would suggest that early Austrodyptornithes may have relied on specialized tactile-foraging methods, somewhat analogous to ancestral palaeognaths being remote-touch probe foragers [2].

Outside Austrodyptornithes, our data indicate that there may be additional examples of unusual neurovascular morphology in the bill tips of other groups of birds, specifically Suliformes (gannets and kin), Eurypygimorphae (tropicbirds, sunbittern and kagu) and Ardei (non-ibis Pelecaniformes). In the context of our new data on Austrodyptornithes, these observations could suggest that a bill-tip organ is plesiomorphic for all Aequorlitornithes or Phaethoquornithes (major aquatic bird clades branching from nodes 7 and 8, figure 2*a*). It is also possible that the widespread presence of a bill-tip organ across Neoaves represents the plesiomorphic retention of a tactile bill-tip organ from an even deeper point in crown bird evolutionary history as has been previously hypothesized [2]. Alternatively, multiple convergent acquisitions of bill-tip organs may have arisen in Neoaves (non-galloanseran Neognathae). Confident resolution of these alternative hypotheses will necessitate investigations of early fossil crown birds and crownward stem birds on a large scale.

## 5. Conclusions

We describe a previously undocumented bill-tip organ in austrodyptornithine seabirds, present in the bills of albatrosses and penguins. This organ may play a role in foraging in low-light conditions underwater, or it may be vestigial, inherited from earlier common ancestors reliant on specialized tactile-foraging techniques. Though it may have evolved independently in albatrosses and penguins, we contend that the bill-tip organ is likely to represent the ancestral state for crown group Austrodyptornithes. If it is used in a sensory capacity in extant Austrodyptornithes, determining its function (particularly in relation to foraging) would be valuable in improving our understanding of seabird foraging ecology, which may have significant implications for their conservation. Our broad sample of extant birds indicates that there may be other examples of bill-tip organs in extant birds and that our understanding of the ecological and evolutionary significance of the tactile organs in bird bills remains relatively incipient.

## Data Availability

The data that support the findings of this study are openly available on the Dryad Digital Repository [78]. Supplementary material is available online [79].
